# Acute gastric volvulus in operated cases of tracheoesophageal fistula

**DOI:** 10.4103/0971-9261.69139

**Published:** 2010

**Authors:** Milind Joshi, Sandesh Parelkar

**Affiliations:** Department of Paediatric Surgery, SETH G.S.M.C. and K.E.M. Hospital, Mumbai, India

**Keywords:** Acute gastric volvulus, complications, esophageal atresia, tracheoesophageal fistula

## Abstract

A report of two neonates of esophageal atresia with tracheoesophageal fistula who had acute gastric volvulus in the postoperative period and required gastropexy after correction of the volvulus. Such postoperative complication has not been reported in the literature so far.

## INTRODUCTION

Congenital esophageal atresia and tracheoesophageal fistula (EA/TEF) is commonly associated with other congenital anomalies. [1] Gastric volvulus can present as an acute abdomen in the infancy. [2–6] However, secondary acute gastric volvulus in the operated tracheoesophageal fistula has not been reported so far.

## CASE REPORTS

### Case 1

A full-term male neonate was operated for EA/TEF (Type C). On the 4^th^ postoperative day, he had sudden epigastric distension and hematemesis. After adequate resuscitation, surgical exploration, and reduction of the gastric volvulus, a gastropexy was done. There was no other pathology detected in the stomach that would predispose it to volvulus.

### Case 2

**Figure 1:**
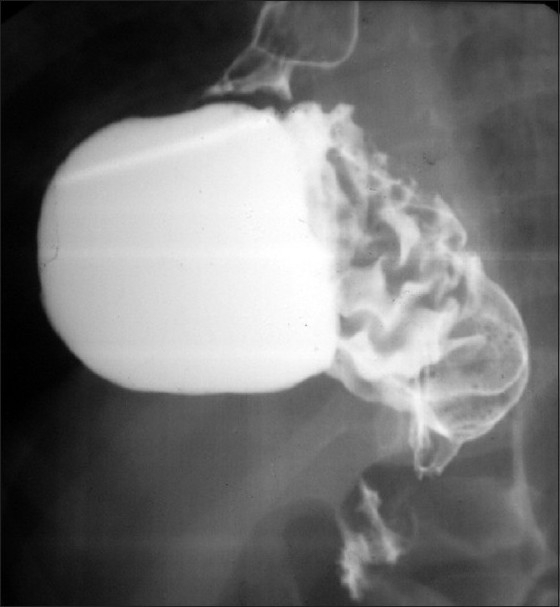
Upper GI contrast study showing developing organoaxial gastric volvulus with dilated stomach with Ryle’s tube *in situ*

This was again a full-term neonate operated for EA/TEF repair. On the 5^th^ postoperative day he developed sudden onset epigastric distension. Abdominal radiographs revealed dilated stomach shadow. Upper gastro-intestinal contrast examination revealed organoaxial volvulus of the stomach [Figure 1]. Exploratory laparotomy revealed similar findings as that of the first case and similar surgery was done. The child was thriving at 1-year follow-up.

## DISCUSSION

In EA/TEF, the incidence of associated congenital anomalies is 50%–70%[1] and gastrointestinal anomalies are reported to be seen in 24% of the cases.[2] Acute gastric volvulus frequently presents in children. Most case reports and the largest single institutional study identify neonates and infants younger than 6 months as the most common age group of children with acute gastric volvulus of the stomach.[3]

There have been less than 600 reported cases of gastric volvulus in children till 2008.[4] The normal stomach is fixed and prevented from volvulus by the ligamentous attachments of the stomach as gastrohepatic, gastrocolic, gastrophrenic and gastrosplenic ligaments. The relative fixity of the pylorus and gastroesophageal junction also helps to maintain the normal position of the stomach.

The volvulus of the stomach is primary when these ligamentous attachments are poor or absent. It can also occur in the presence of normal ligamentous attachments.[3] It can also be secondary to congenital diaphragmatic hernia, hiatus hernia, diaphragmatic eventration, paraesophageal hernia, wandering spleen, with distended stomach and gastric outlet obstruction or with malrotation of the intestine.[5,6] It may also occur secondary to Nissen’s fundoplication.[7] The volvulus can be of organoaxial, mesentericoaxial, or mixed type depending on the axis on which the stomach rotates onto itself.[8] The organoaxial type is more commonly found in the primary type.[9] Most of the mesentericoaxial volvulus in children is secondary to diaphragmatic hernia and paraesophageal hernia.[4] The diagnosis is usually by clinical suspicion in the setting of underlying pathology and by upper GI contrast study. Gastropexy is the commonly preferred surgery either open or laparoscopically.[10]

Both our cases reported here were diagnosed to be having EA/TEF in their routine postnatal evaluation. Both the patients had large TEF and the esophageal repair had been done after the mobilization of the upper and lower esophageal pouch and had uneventful perioperative course. Both the patients were expected to have good recovery from the TEF when this unexpected pathology struck on postoperative day 4 and day 5, respectively. In the first case, the neonate developed sudden onset epigastric distension and hematemesis. Roentgenogram of the abdomen and chest was normal. But because of the suspicion of the gastric volvulus, exploration was done, which revealed the organoaxial gastric volvulus with normal attachments and no other pathology. The esophageal anastomosis was also intact, which was confirmed on dye study after the correction of the gastric volvulus.

The second neonate developed same symptoms on the 5^th^ postoperative day, with abdomen radiograph showing large stomach shadow and the presence of epigastric lump, hence upper GI dye study was done to ascertain the diagnosis of gastric volvulus. It confirmed the organoaxial type of the volvulus. Emergency exploration and three-point gastropexy were done. The patient made uneventful recovery from both the surgeries and was doing well at one year of follow up.

The literature on gastric volvulus mentions secondary gastric volvulus as more common; however, TEF does not figure in the list of etiology.[3] The occurrence of gastric volvulus in our patients was really surprising and no definite explanation can be given. We do not know whether it is a mere coincidence of the two pathologies or it could be attributed to the preferential passage of air into the stomach in the postnatal period before the ligation of the large TEF leading to the stretching of the supporting ligaments and predisposing it for volvulus. Another reason could be the esophageal anastomosis stretching the esophagogastric junction and acting as a fixed point causing stomach volvulus. The transanastomotic tube placed in the stomach for free drainage has also not helped to prevent the pathology. It won’t be overemphasizing here that the pathogenesis of the mechanisms of volvulus described for both types was observed to be occurring simultaneously in our cases leading to the organoaxial volvulus in the absence of ligamentous abnormality and TEF acting as a predisposing factor. However, the incidence of this pathology may appear as mere coincidence because of the rarity of its occurrence.

We feel that although not reported till now, gastric volvulus can occur in operated cases of the TEF and prompt management should be done if the pathology is suspected. This is the first report of such gastric volvulus occurring in the setting of TEF.
